# Antidepressant Paroxetine Exerts Developmental Neurotoxicity in an iPSC-Derived 3D Human Brain Model

**DOI:** 10.3389/fncel.2020.00025

**Published:** 2020-02-21

**Authors:** Xiali Zhong, Georgina Harris, Lena Smirnova, Valentin Zufferey, Rita de Cássia da Silveira e Sá, Fabiele Baldino Russo, Patricia Cristina Baleeiro Beltrao Braga, Megan Chesnut, Marie-Gabrielle Zurich, Helena T. Hogberg, Thomas Hartung, David Pamies

**Affiliations:** ^1^Center for Alternatives to Animal Testing (CAAT), Johns Hopkins Bloomberg School of Public Health, Baltimore, MD, United States; ^2^Guangdong Provincial Key Laboratory of Food, Nutrition and Health, Department of Toxicology, School of Public Health, Sun Yat-sen University, Guangzhou, China; ^3^Department of Physiology, Lausanne and Swiss Centre for Applied Human Toxicology (SCAHT), University of Lausanne, Lausanne, Switzerland; ^4^Department of Physiology and Pathology, Federal University of Paraíba, João Pessoa, Brazil; ^5^Department of Microbiology, Institute of Biomedical Sciences, University of São Paulo, São Paulo, Brazil; ^6^Department of Obstetrics, School of Arts Sciences and Humanities, São Paulo, Brazil; ^7^CAAT-Europe, University of Konstanz, Konstanz, Germany

**Keywords:** paroxetine, SSRI, organoid, neurotoxicity, developmental neurotoxicity, 3D, iPSC

## Abstract

Selective serotonin reuptake inhibitors (SSRIs) are frequently used to treat depression during pregnancy. Various concerns have been raised about the possible effects of these drugs on fetal development. Current developmental neurotoxicity (DNT) testing conducted in rodents is expensive, time-consuming, and does not necessarily represent human pathophysiology. A human, *in vitro* testing battery to cover key events of brain development, could potentially overcome these challenges. In this study, we assess the DNT of paroxetine—a widely used SSRI which has shown contradictory evidence regarding effects on human brain development using a versatile, organotypic human induced pluripotent stem cell (iPSC)-derived brain model (BrainSpheres). At therapeutic blood concentrations, which lie between 20 and 60 ng/ml, Paroxetine led to an 80% decrease in the expression of synaptic markers, a 60% decrease in neurite outgrowth and a 40–75% decrease in the overall oligodendrocyte cell population, compared to controls. These results were consistently shown in two different iPSC lines and indicate that relevant therapeutic concentrations of Paroxetine induce brain cell development abnormalities which could lead to adverse effects.

## Introduction

Between 7 and 12% of pregnant women suffer from depression (Bennett et al., 2004). Selective serotonin reuptake inhibitors (SSRIs) are one of the most commonly used treatments (Andrade et al., 2008; Alwan et al., 2011). Several concerns about the possible developmental neurotoxicity (DNT) effects of different SSRIs have been raised over the years (i.e., antidepressants such as fluoxetine, paroxetine, citalopram, and sertraline). Indeed, neurobehavioral studies involving SSRIs have shown adverse effects on neonates (Zeskind and Stephens, 2004; Alwan and Friedman, 2009; Gentile and Galbally, 2011), infants and young children.

Paroxetine was shown to cross the placental barrier (Hendrick et al., 2003) and was often the center of attention for possible adverse effects (Nevels et al., 2016), including autism (Posey et al., 1999; Harrington et al., 2013; Boukhris et al., 2016). Croen et al. (2011) followed 145,456 full-term infants for a total of 904,035 person-years; they reported increased risk from 1% to 1.87%, with a 95% CI of 1.15–3.04, but several shortcomings of the study were noted (Croen et al., 2011; Nevels et al., 2016). A systematic review in 2016 (Boukhris et al., 2016) reported an odds ratio of 2.13 (95% CI: 1.66–2.73) for developing autism in children prenatally exposed to SSRIs compared to an odds ratio of 1.81 (95% CI: 1.47–2.24) in those unexposed.

The use of paroxetine in pregnancy has declined substantially (Meunier et al., 2013) due to the US FDA warning in 2005 regarding the potential risk for cardiac defects in the fetus (Cole et al., 2007) and some evidence of major congenital malformations, especially in children (Berard et al., 2016; Gao et al., 2018). However, the effects are not clear and there are contradictory results (Ellfolk and Malm, 2010; Alwan et al., 2016). Even though the use during the first trimester is contraindicated, paroxetine is still used later in pregnancy and during breastfeeding. To the best of our knowledge, there are no studies that explore the consequences of long-term exposure of the developing brain to SSRIs. In this project, we aim to study the possible deleterious effects of the SSRI paroxetine may exert on different key processes during brain development.

DNT is of high concern, however, no routine testing for DNT is carried out in any regulatory program worldwide. Indeed, DNT testing is not required unless triggered by the observation of neurotoxic or endocrine effects in adult rodents. Furthermore, as described in the OECD guidelines, DNT experiments are also extremely expensive (1.4 million per substance), as well as time- and animal-consuming (1,400 pups per compound). Moreover, human brain complexity may not be completely reflected in animal models. The same shortcomings apply for toxicity testing of drugs developed in the pharmaceutical industry. Thus, thousands of drugs and chemicals reach the market without proper classification regarding DNT.

There is consensus in the field that more reliable and efficient screening and assessment tools are required for better identification and evaluation of DNT chemicals and drugs. Over the last 15 years, there has been a process to develop an *in vitro* testing battery to cover key events of neurodevelopment, such as neural stem cell proliferation and differentiation, migration, neurite outgrowth, synaptogenesis, neuronal network formation, myelination, and apoptosis (Bal-Price et al., 2012; Smirnova et al., 2014). Furthermore, the use of more human-relevant models, based on 3D organotypic induced pluripotent stem cell (iPSC)-derived systems, has been recommended as an alternative to classical *in vitro* models (Bal-Price et al., 2012; Fritsche et al., 2018a,b; Smirnova et al., 2018).

The previously described 3D human iPSC-derived brain model (BrainSpheres) recapitulates some of the key events of neurodevelopment (Pamies et al., 2017). BrainSpheres are very reproducible in terms of size and cellular composition and do not display necrotic centers. They not only contain neurons and astrocytes but also functional oligodendrocytes with 40–50% axonal myelination, which is rarely observed *in vitro* (Pamies et al., 2017). In this study, we used the BrainSphere model to study the effects of paroxetine on different processes of brain development. Exposure to human-relevant therapeutic blood concentrations of paroxetine (Tomita et al., 2014) led to alterations in synaptic markers expression, myelination, neurite outgrowth and oligodendrocyte numbers in BrainSpheres differentiated from two independent iPSCs lines, strongly suggesting paroxetine as a DNT toxicant.

## Materials and Methods

### Chemicals and Exposure

Paroxetine was supplied by Sigma. A stock of 10 μg/ml was prepared in DMSO Hybri-Max (Sigma) and stored at −20°C. DMSO (0.072%) was used as vehicle control to match the amount of DMSO in the highest paroxetine concentration of 60 ng/ml.

### BrainSphere Differentiation

The CRL-2097 line was derived from CCD-1079Sk ATCC^®^ CRL-2097™ fibroblasts purchased from ATCC and was kindly provided by Dr. Hongjun Song within our joint NIH NCATS funded project (Pamies et al., 2017; #1U18TR000547-01). The iPS2C1 line was kindly provided by Dr. Herbert Lachman. All studies followed Institutional Review Board protocols approved by the Johns Hopkins University School of Medicine. Differentiation from iPSCs to NPCs has been previously described (Wen et al., 2014). The BrainSpheres were generated as described in Pamies et al. (2017). Briefly, at 90% confluency, NPCs were detached mechanically and counted. The 2 × 10^6^ cells per well were plated in uncoated six well-plates. After 2 days, NPC medium was changed to differentiation medium (Neurobasal^®^ electro Medium (Gibco) supplemented with 2% B-27^®^ Electrophysiology (Gibco), 1% Glutamax (Gibco), 0.01 μg/ml human recombinant GDNF (Gemini), 0.01 μg/ml human recombinant BDNF (Gemini). Cultures were kept at 37°C in an atmosphere of 5% CO_2_ under constant gyratory shaking (88 rpm, 19 mm orbit) for up to 8 weeks. The medium was partly exchanged three times a week.

### Cell Viability

Cytotoxicity to BrainSpheres was assessed after exposure to 0, 20 and 60 ng/ml of paroxetine continuously for 8 weeks. After drug exposure, resazurin reduction assay was performed. One-hunderd microliter of 2 mg/ml Resazurin were added directly to 6-well plates (2 ml/well). The plates were incubated for 3 h at 37°C, 5% CO_2_. Subsequently, 50 μl of medium from each well were transferred to 96-well plates and the fluorescence of resorufin was measured at 530 nm/590 nm (excitation/emission) using a multi-well fluorometric reader CytoFluor series 4000 (PerSeptive Biosystems, Inc., Framingham, MA, USA). To determine statistical significance, an one-way ANOVA test was performed with *post hoc* Bonferroni test. All data given are the means ± SD of three independent experiments performed with three technical replicates in both cell lines.

### Mitochondrial Membrane Potential Assay

Mitochondrial dysfunction was measured by MitoTracker Red CMXRos (Life Technologies, Carlsbad, CA, USA) following the protocol described in Pamies et al. (2018). Briefly, after 8 weeks of exposure to 0, 20 or 60 ng/ml of paroxetine, 10 BrainSpheres per condition were plated in 24-well-plates (500 μl). One microliter of MitoTracker Red CMXRos was added to the medium and incubated for 30 min at 37°C, 5% CO_2_. The BrainSpheres were then washed twice and fixed with 4% paraformaldehyde (PFA) for 1 h and washed again twice with PBS. The Shandon Immuno-Mount (Thermo Fisher Scientific, Waltham, MA, USA) was used to mount the spheroids onto microscope cover slides (Thermo Fisher Scientific, Waltham, MA, USA). Images were taken using a Olympus BX60. The fluorescence was quantified using ImageJ software^1^ and normalized to the size of the aggregates. To determine statistical significance, one-way ANOVA was performed with *post hoc* Bonferroni test. All data given are the means ± SD of three independent experiments performed with 10 technical replicates.

### Immunohistochemistry

BrainSpheres were collected at 8 weeks of differentiation, washed three times for 5 min with PBS and fixed with 4% PFA for 1 h at room temperature followed by two washing steps with PBS. BrainSpheres were incubated for 1 h in blocking solution consisting of 5% normal goat serum (NGS) in PBS with 0.4% Triton-X100 (Sigma). BrainSpheres were then incubated for 48 h at 4°C with a combination of primary antibodies (Table 1) diluted in PBS containing 3% NGS and 0.1% Triton-X100. After this incubation, BrainSpheres were washed three times for 15 min in 1× PBS and incubated 1 h with secondary antibodies (Table 1) diluted in PBS with 3% NGS at room temperature. Subsequently, BrainSpheres were washed three times for 5 min each with PBS, the nuclei were stained with Hoechst 33342 (1:10,000, Thermo Fisher Scientific, Waltham, MA, USA) for 60 min. BrainSpheres were mounted on glass slides by using Shandon Immu-mount. The images were taken using a Zeiss UV-LSM 510 confocal microscope and a Zeiss LSM 780 GaAsP.

**Table 1:** Primary antibodies for immunohistochemistry.

Antibody	Host	Type	Source	Dilution
NF200	Rabbit	Polyclonal	Sigma	1:200
GFAP	Rabbit	Polyclonal	Dako	1:500
βTUBIII	Rabbit	Polyclonal	R&D	1:200
S100β	Rabbit	Polyclonal	Santa Cruz	1:200
O4	Mouse	Monoclonal	R&D	1:200
O1	Mouse	Monoclonal	Millipore	1:500
MBP	Mouse	Monoclonal	Biolegend	1:200
Synaptophysin	Mouse	Monoclonal	Sigma	1:200
PSD95	Rabbit	Monoclonal	Life technologies	1:200
Secondary antibodies for immunohistochemistry
Alexa Fluor 488 goat anti-mouse			Life Technologies	1:500
Alexa Fluor 658 goat anti-rabbit			Life Technologies	1:500

### Neuronal Synaptic Pixel Quantification

After 8 weeks of differentiation, BrainSpheres were fixed and stained for synaptophysin (SYP, pre-synaptic protein; Table 1), along with Neurofilament Protein (NF200; Table 1), for cell identification. In addition, the same final cell density was confirmed by Hoechst staining for each condition. Immunofluorescent images were taken randomly for each condition at 63× and SYP pixels were quantified after three-dimensional reconstruction of z-stack confocal images considering the number of pixels for neurite length, using ImageJ. Quantification was performed blindly.

### Western Blot Analysis

BrainSpheres were collected after paroxetine exposure for 8 weeks. One-hundred and fifty micro liter RIPA lysis buffer (Sigma–Adrich, St. Louis, MO, USA) containing 1× protease inhibitor cocktails (Sigma–Adrich, St. Louis, MO, USA) was added to each sample and incubated on ice for 30 min to lyse the cells. Then the samples were centrifuged at 12,000 rpm, 4°C for 15 min, the supernatant was transferred to a new tube and incubated with pierce™ lance marker reducing sample buffer (Thermo Fisher Scientific, Waltham, MA, USA) at 95°C for 5 min. The protein concentration was measured with the pierce™ BCA protein assay kit (Thermo Fisher Scientific, Waltham, MA, USA). A total of 30 μg protein was separated on a 4–15% gradient SDS-polyacrylamide gel with 80 V for 120 min and transferred to a polyvinylidene difluoride membrane by electroblotting with 200 mA on ice for 2 h. The non-specific membrane binding was blocked with a blocking solution (PBS, 0.5% Tween-20, 5% non-fat dry milk, pH 7.4) for 1 h at room temperature. Subsequently, the membrane was incubated with primary antibodies Synaptophysin, 1:800, Sigma–Adrich, St. Louis, MO, USA; PSD95, 1:1,000, Thermo Fisher Scientific, Waltham, MA, USA; GAPDH, 1:1,000, Cell Signaling Technology, Danvers, MA, USA) in blocking solution overnight at 4°C. The membrane was washed thoroughly with PBS-T and incubated with HRP-conjugated goat anti-mouse or goat-anti-rabbit secondary antibodies anti-mouse, 1:3,000, BIO-RAD; anti-rabbit, 1:2,000, Cell Signaling Technology, Danvers, MA, USA)in blocking solution at room temperature for 1 h. The blotting bands were detected by chemiluminescence reagent plus (BIO-RAD, Hercules, CA, USA), and exposed to the X-ray film.

### Neurite Outgrowth and Astrocytes Staining

BrainSpheres were cultivated as described above. After 8 weeks of exposure to 0, 20 or 60 ng/ml of paroxetine, BrainSpheres were seeded on Matrigel™ (BD Biosciences) pre-coated, flat-bottom, black 24-well plates (Thermo Fisher Scientific, Waltham, MA, USA). After 24 h, the BrainSpheres were fixed in 4% PFA, stained with anti-β-III-Tubulin (neuronal marker) and GFAP (astrocytes marker) as described above and imaged using a confocal microscope Zeiss UV-LSM 510 and analyzed using the Sholl ImageJ Software^2^. For data analysis, the number of intersections/distance from spheroid center was calculated and the mean plotted. Significance was calculated by using the Area Under the Curve. ANOVA with Dunnett’s post-test was performed comparing each treatment with the control. **P < 0.01. Intersections were counted after 300 μm diameter (average size of the spheroids).

### Oligodendrocyte Quantification

BrainSpheres were exposed for 8 weeks to 0, 20 or 60 ng/ml of Paroxetine, fixed with 4% PFA and stained with anti-O4 antibody. Immunohistochemistry was performed as described above. O4-positive cells were counted in four different experiments by four different individuals, median and standard deviation (SD) were calculated from the count of each individual.

## Results

### Therapeutic Concentrations of Paroxetine Do Not Alter Cell Viability, Mitochondrial Function and Neuronal/Astrocyte Phenotype

**Figure 1:**
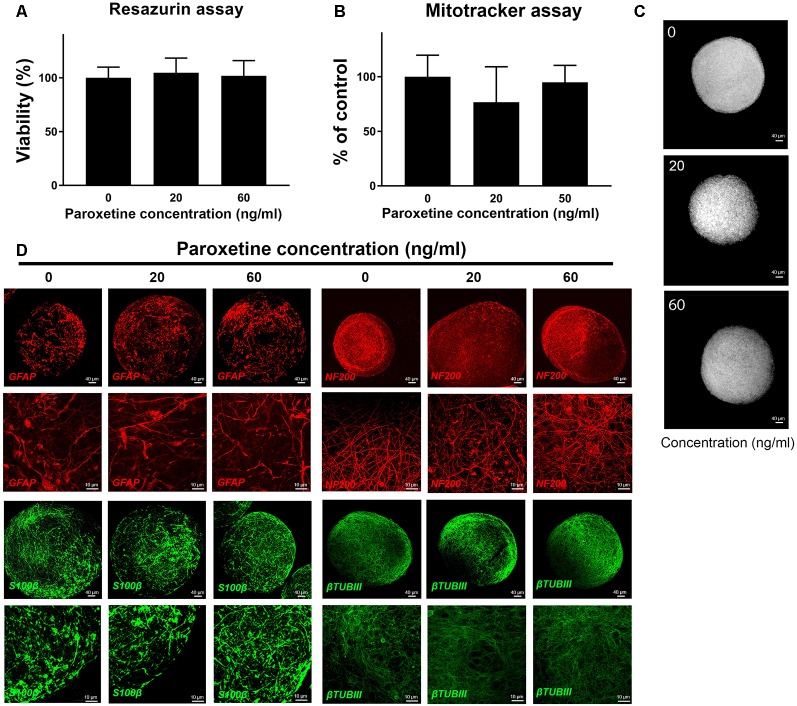
Cell viability, mitochondria function and immunohistochemistry of BS after exposure to paroxetine. BrainSpheres were treated during the differentiation process (8 weeks) with 20 or 60 ng/ml paroxetine. This is a representative figure of the experiment performed, both cell lines have shown similar results. **(A)** Percentage of viable cells in paroxetine-treated BrainSpheres, normalized to the vehicle control as measured by resazurin assay. Data are presented as mean ± SD from three independent experiments. **(B)** Mitochondrial membrane potential measured (MMP) by mitotracker assay. Vehicle-treated control was used to set 100% MMP. **(C)** Representative images of mitotracker assay. **(D)** Immunohistochemistry with astrocyte markers (GFAP, S100β) and neuronal markers (NF200, βTUBIII).

To determine, if therapeutic concentrations could produce general cytotoxicity, resazurin assay, and mitotracker analysis were performed after 8 weeks of exposure to paroxetine (0, 20 and 60 ng/ml) in two cell lines (Figures 1A,B and data not shown). No significant difference in cell viability and mitochondrial membrane potential (Figure 1C) was observed at the concentrations studied. Immunohistochemistry for astrocyte markers (S100β and GFAP) and neuronal markers (NF200 and βTUBIII) also showed no changes upon exposure to paroxetine (Figure 1D).

### Paroxetine Exposure Alters the Expression of Synaptic Markers in BrainSpheres

**Figure 2:**
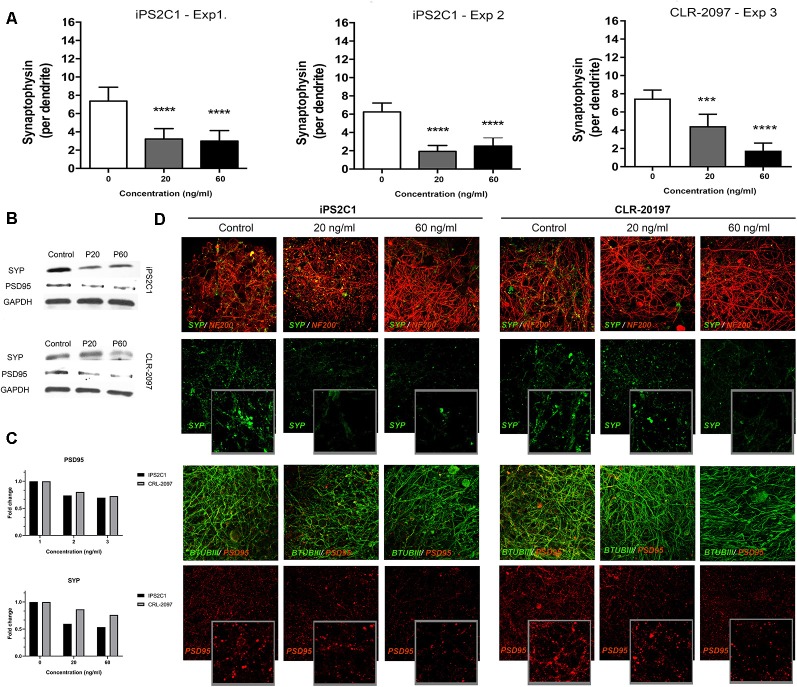
Synaptic markers analysis after paroxetine exposure. BrainSpheres were exposed to paroxetine (0, 20 or 60 ng/ml) for 8 weeks of differentiation. After 8 weeks BrainSpheres were collected to perform immunohistochemistry and Western blot. **(A)** Blinded quantification of synaptophysin (SYP) pixels after three-dimensional reconstruction of z-stack confocal images from three different experiments (two for iPS2C1 and one for CLR-2097). At least 10 spheroids were imaged for each experiment. **(B)** Western blot analyses of SYP, PSD95, and GAPDH. **(C)** Densitometry of western blot analysis. **(D)** Representative images for synaptic markers. Upper panel: SYP (green) co-stained with neuronal marker NF200 (red); lower panel: postsynaptic marker PSD95 (red) co-stained with neuronal marker βTUBIII (green). ***P < 0.01; ****P < 0.001.

BrainSpheres were exposed to therapeutic-relevant paroxetine concentrations (Tomita et al., 2014) for the 8 weeks of differentiation. After 8 weeks of treatment, BrainSpheres were collected, fixed and stained with different antibodies as described in materials and methods. SYP quantification showed a statistically significant decrease in this marker in BrainSpheres generated from both iPSC lines (Figure 2A). In the iPS2C1 line, a 60 and 70% decrease in SYP staining was observed, at both concentrations. The CLR-2097 line showed a dose-dependent reduction of approximately 40 and 80%, at 20 and 60 ng/ml, respectively (Figure 2A). Western blot results confirmed the decrease in SYP and PSD95 markers in both iPSC lines (Figures 2B,C). By western blot, a stronger effect on SYP levels was observed in the iPS2C1 line. The CLR-2097 line showed a dose-dependent decrease in SYP, similar to the immunohistochemistry quantification results (Figures 2A,B). Paroxetine exposure also decreased a post-synaptic marker (PSD95) in both cell lines but to a lesser extent than SYP, as shown by immunohistochemistry (Figure 2D). These results show a consistent decrease in SYP and PSD95 markers after paroxetine exposure which may result in adverse effects on synaptogenesis during neural differentiation.

### BrainSpheres Neurite Outgrowth Capability Is Reduced After Paroxetine Exposure

**Figure 3:**
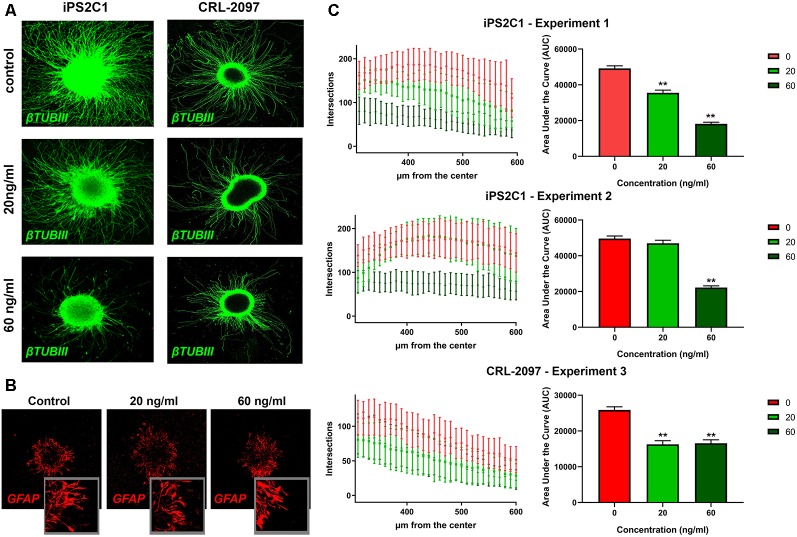
Neurite outgrowth analysis after paroxetine exposure. BrainSpheres were cultivated for 8 weeks with and without paroxetine (20 or 60 ng/ml). After treatment, cells were seeded on Matrigel^®^ for 24 h and fixed for immunohistochemistry. **(A)** βTUBIII staining of attached BrainSpheres after 24 h for both cell lines. **(C)** Sholl ImageJ neurite outgrowth quantification for a total of three experiments in two different cell lines. In the left panel the X-axis represents radius from the center while the Y-axis represents the number of intersections with the concentric circles produced by the software. In the right panel, the area under the curve is shown for the three experiments. The red line represents vehicle control; light green represents 20 ng/ml of paroxetine; dark green represents 60 ng/ml paroxetine treatment. **(B)** Immunohistochemistry with anti-GFAP antibody on attached spheres (24 h) for iPS2C1 line after 8 weeks of treatment with the different concentrations. Statistical analysis was performed by using ANOVA with Dunnett’s post-test comparing treated with control (untreated). **P < 0.01.

BrainSpheres were cultivated for 8 weeks with and without the presence of paroxetine (20 or 60 ng/ml). In order to quantify neurite outgrowth, BrainSpheres were attached to Matrigel-coated 24-well plates after 8 weeks of exposure to paroxetine and cultured for further 24 h. Neurite outgrowth analysis showed a consistent statistically significant decrease in neurite density in both cell lines treated with 60 ng/ml of paroxetine in different experiments (Figures 3A,C). The iPS2C1 line showed a higher number of neurites and in consequence a higher number of intersections (Figure 3A). iPS2C1-derived BrainSpheres presented reproducibility across experiments with around 187 ± 35 intersections 400 μm from the BrainSphere center in experiment 1 and 178 ± 39 intersections 410 μm from the BrainSphere center in experiment 2. Cells treated with 60 ng/ml presented a 60% decrease in the number of intersections (neurites). However, some variability was found at 20 ng/ml exposure (light green, Figure 3C); leading to a statistically significant dose-dependent decrease in number of neurites in experiment 1 and no effects compared to control in experiment 2 (Figure 3C). In the CRL-2097 line, BrainSpheres presented a maximum of intersections 114 ± 26 at 290 μm from the center, with a 25% decrease in the number of neurites at both 20 and 60 ng/ml. The area under the curve was used to compare treatments with controls, showing a consistent decrease in number of neurites at 60 ng/ml and significant change in two of three experiments at 20 ng/ml paroxetine treatment (Figure 3C). Since we observed that 20 ng/ml paroxetine reduced neurite outgrowth in two out of three experiments performed in two lines, and 60 ng/ml paroxetine reduced neurite outgrowth consistently in all experiments, we concluded that paroxetine at therapeutic concentrations has the potential to affect neurite outgrowth during brain development. Additionally, no changes were observed in astrocyte morphology by immunostaining after treatments with paroxetine (Figures 1C, 3B).

### Paroxetine Affects Oligodendrocyte Population

**Figure 4:**
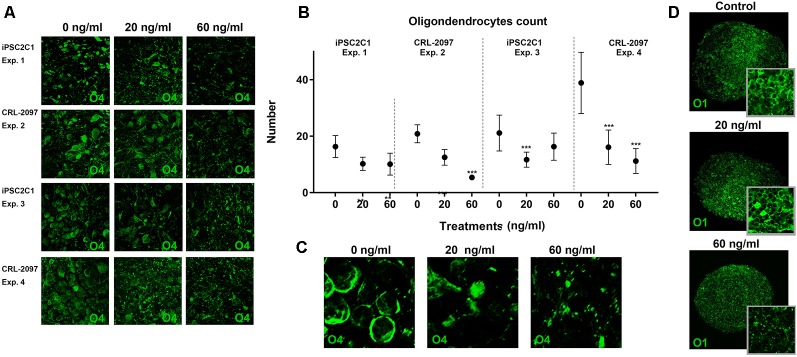
Quantification of oligodendrocytes population. BrainSpheres were cultivated for 8 weeks with and without the presence of paroxetine (0, 20 or 60 ng/ml). After treatment, spheres were fixed for immunohistochemistry. **(A)** O4 staining of BrainSpheres from both cell lines (two independent experiments per line). **(B)** O4-positive cells quantification from four experiments.** (C)** Blow-up of a representative 04 staining. **(D)** O1 staining of BrainSpheres from CLR-2097 line. Statistical analysis was performed by using ANOVA with Dunnett’s post-test comparing treated with control (untreated). ***P < 0.01.

BrainSpheres were cultivated for 8 weeks in the presence and absence of paroxetine (0, 20 or 60 ng/ml). After 8 weeks of treatment, BrainSpheres were collected, fixed and stained with different antibodies as described in materials and methods (Figures 4A,C). Although O1 is considered a marker for mature oligodendrocytes and O4 a marker for immature oligodendrocytes, both antibodies presented a similar pattern within BrainSpheres (Figures 4A,D). This co-expression of O4 and O1 has been described by several authors (Silbereis et al., 2010; Fröhlich et al., 2011; Traiffort et al., 2016). The fact that cells in this model still express O4 indicates that in the BrainSpheres, oligodendrocytes do not reach full maturation within 8 weeks. Since, O4 presented better cell body definition and less background immunostaining, it was selected for oligodendrocyte quantification in four independent experiments that were performed, two per cell line. Confocal images for O4 (Supplementary Figure S1) were blindly quantified by four different experimenters and represented graphically (Figure 4B). The results showed a statistically significant decrease of O4-positive cells in all BrainSpheres treated with paroxetine except in the second experiment using the iPS2C1 line treated with 60 ng/ml paroxetine where the observed decrease was not significant. Myelination of axons was quantified in one independent experiment (10 replicates) as described in material and methods and was decreased in paroxetine-treated BrainSpheres (Supplementary Figure S2). A decrease in myelination was observed in further three experiments with both cell lines, however, were not quantified due to noisy staining with the MBP antibody.

## Discussion

Paroxetine, a SSRI is contraindicated during the first trimester of pregnancy mainly because of the increased risk of cardiac and other congenital malformations (Cole et al., 2007). However, this drug is still used after this period (second and third trimester) as well as during breastfeeding (Orsolini and Bellantuono, 2015). Very few studies can be found addressing neurobehavioral effects after chronic prenatal exposure to paroxetine, however, negative effects have been reported in neonates (Zeskind and Stephens, 2004; Alwan et al., 2007; Gentile and Galbally, 2011; Klinger et al., 2011), infants and young children (Casper et al., 2003, 2011; Oberlander et al., 2005, 2010; Klinger et al., 2011; Harrington et al., 2013; Rai et al., 2013). Rat studies have shown that pharmacological or genetic modifications of serotonin levels in the developing brain produce adverse effects on adult emotional behavior (Lisboa et al., 2007; Olivier et al., 2011; Glover et al., 2015; Glover and Clinton, 2016; Zohar et al., 2016). In addition, studies in infants whose mothers were treated with paroxetine during breastfeeding have shown deficits in alertness, sleepiness, irritability, as well as low body temperature, uncontrollable crying, eating and sleeping disorders (Costei et al., 2002; Hale et al., 2010; Uguz and Arpaci, 2016; Uguz, 2018). However, it has remained a challenge to correlate these symptoms with exposure to paroxetine during development (National Library of Medicine, 2006).

First and early second trimesters of pregnancy are vital for the development of the heart (Mäkikallio et al., 2005; Valenti et al., 2011). Serotonin plays an important role in heart formation and has been reported to be involved in the regulation of proliferation in the embryonic heart (Frishman and Grewall, 2000; Nebigil et al., 2000, 2003; Nebigil and Maroteaux, 2001). Deregulation of this developmental process by the excess of serotonin due to paroxetine treatment during the first trimester of pregnancy may explain cardiac malfunction. The much longer duration needed for proper brain development, which extends until adolescence (Epstein, 1986), increases the period of vulnerability of the brain to developmental toxins.

Serotonin plays an important role in cognitive processes, such as memory and learning (Berridge et al., 2009) and is crucial during brain development (Buznikov et al., 2001). Therefore, subtle modulation of serotonin levels by paroxetine during brain development may have important deleterious consequences later in life. Manipulations of serotonin levels in rodent brains during early development were shown to alter the formation of the whisker (barrel) representation in the primary somatosensory cortex and promote aggressive and/or anxiety-related behaviors (Cases et al., 1995; Persico et al., 2000, 2001; Holmes et al., 2003). Behavioral changes were also observed when serotonin levels are modified in rodents’ early-life (Welker et al., 1996; Yang et al., 2001; Esaki et al., 2005).

Effects of paroxetine on key processes of brain development have to be established in order to evaluate its potential DNT. However, current DNT testing is facing numerous challenges. DNT experts have raised concerns about the relevance of animal data for human risk assessment and have recommended substituting the expensive and time-consuming rodent guidelines for an *in vitro* testing battery comprising human-relevant models such as 3D organo-typic iPSC-derived systems (Bal-Price et al., 2012), covering key events of neurodevelopment. The goal of this study was to establish a battery to help the identification of DNT compounds. Here, we took advantage of our 3D iPSC-derived human *in vitro* model, the BrainSpheres, enabling the study of various key events, such as a neuron, astrocyte and oligodendrocyte differentiation and maturation, neurite outgrowth, synaptogenesis, and myelination, to study the potentially deleterious effects of paroxetine. Our model allows performing multiple assays covering different key events in a single model system facilitating its applicability. For this study, relevant, therapeutic-blood concentrations of 20 and 60 ng/ml paroxetine (Tomita et al., 2014) were chosen. BrainSpheres were exposed during the entire differentiation process. In order to show robust results, we decided to use two different iPSC lines to generate the BrainSpheres and used at least three independent experiments per assay. Between 5 and 10 technical replicates (spheroids) were analyzed for each experiment.

BrainSpheres exposed to 20 or 60 ng/ml paroxetine for 8 weeks did not present any cytotoxic effects or mitochondrial dysfunction (Figures 1A,B) in either of the lines studied. Moreover, immunohistochemistry for astrocytic markers (GFAP and S100β) and neuronal markers (NEF200 and BTUBIII) did not show any changes after paroxetine treatment (Figure 1D). However, our functional assays showed some DNT effects produced by paroxetine exposure, in line with different animal studies indicating that serotonin or 5-hydroxytryptamine (5-HT), together with other neurotransmitters, is implicated in developmental processes such as proliferation, migration, differentiation, and morphogenesis (Buznikov et al., 2001).

Gene expression analysis for the serotonin transporter (SLC6A4; Supplementary Figure S3) showed a decrease after paroxetine exposure, however, this change was not statistically significant. The synaptic marker (SYP) was quantified in BrainSpheres derived from both iPSCs line (iPS2C1 and CLR-2097), showing a consistent statistical significant reduction over the experiments and lines (Figure 2A). We also observed that BrainSpheres derived from the line CLR-2097 were slightly less sensitive to 20 ng/ml paroxetine than BrainSpheres derived from iPS2C1, indicating that studies involving different cell lines might provide insight towards different individual sensitivity to paroxetine effects. These results were also confirmed by Western blot analysis, showing a stronger reduction of SYP in the iPS2C1 line than CLR-2097 (Figures 2A,B). Furthermore, staining for a postsynaptic marker, PSD95, showed a decrease in expression of this protein. These results show a consistent reduction of pre- and postsynaptic markers (SYP and PSD95, respectively) after paroxetine exposure, indicating this antidepressant may affect synaptogenesis during neural differentiation. Animal studies have shown that serotonin depletion during brain development disrupts normal synaptogenesis, producing decreased synaptic density (Mazer et al., 1997). On the other side, the SSRI fluoxetine has been reported to reduce monoamine oxidase gene expression, the primary metabolizing enzyme for serotonin (Bond et al., 2020). Furthermore, some SSRIs have been shown to modulate sodium channels (Thériault et al., 2015; Nakatani and Amano, 2018), which are thought to play a pivotal role during CNS development, since action potential propagation and excitatory transmission are vital for neuronal maturation (Shatz, 1990). Although changes in serotonin levels in the brain of the fetus after maternal exposure to SSRI are not clear, changes in levels of this important neurotransmitter in the brain could have severe consequences on synaptogenesis.

We also observed a statistically significant decrease in neurite outgrowth at 60 ng/ml in all the experiments with both lines, however, 20 ng/ml presented differing results (Figures 3A,C) potentially. CLR-2097 showed practically the same results at 20 and 60 ng/ml, while in iPS2C1 we observed statistically significant changes only in one of two experiments, albeit a decreasing trend (Figure 3C). The differences between the two lines could be due to the higher neurite outgrowth in iPS2C1 than CLR-2097, or because of different sensitivity to paroxetine. It is possible, that 20 ng/ml is close to the threshold that affects this specific endpoint leading to the observed experimental variability (Figures 3A,C). The decrease in neurite outgrowth observed in BrainSpheres after paroxetine exposure is in line with the role of serotonin in this developmental process (Rojas et al., 2014). It is known, that neurotransmitters such serotonin and dopamine are involved in neurite outgrowth and synapse formation (Haydon et al., 1984; Lipton and Kater, 1989; van Kesteren and Spencer, 2003; Daubert and Condron, 2010), therefore, alterations in the level of these neurotransmitters could lead to adverse effects on these key processes. Our data shows disruption on neurite outgrowth and decrease expression of synaptic markers, indicating that changes in serotonin levels may be directly or indirectly responsible for these disruptions (Figures 2, 3).

Oligodendrocyte differentiation and myelin formation are two key events of neural development that have remained difficult to cover in DNT test batteries due to the difficulty to differentiate oligodendrocytes *in vitro*. Myelination is one of the strongest features of the BrainSphere model since this process is rarely observed *in vitro*. Few *in vitro* protocols have been developed recently to obtain oligodendrocytes from human embryonic stem cells or iPSCs (Czepiel et al., 2011; Stacpoole et al., 2013; Wang et al., 2013; Douvaras et al., 2014; Piao et al., 2015; Ehrlich et al., 2017) and other stem cell sources (Najm et al., 2013; Yang et al., 2013). However, to our knowledge, BrainSpheres is one of the few human *in vitro* systems able to produce oligodendrocytes in a 3D model enabling the winding of oligodendrocytes processes around the axons. By using image analysis we were able to show a decreased number of oligodendrocytes accompanied by a decreased expression of MBP (Figure 4 and Supplementary Figure S2) after Paroxetine exposure. In line with our data, previous *in vitro* studies have suggested that an increase of serotonin levels may disrupt oligodendrocytes maturation and myelin formation (Fan et al., 2015). Moreover, exposure to other SSRIs, such as fluoxetine have shown to produce long-term changes in the expression of genes involved in myelination in adult rats (Kroeze et al., 2016). This also correlated with our data on oligodendrocyte quantification (Figure 4) and may indicate that changes in serotonin levels in BrainSphereshave an adverse effect on oligodendrocyte maturation and myelin formation.

In conclusion, some indications from clinical studies suggested that paroxetine may affect brain development, but these results were inconsistent. By using a battery of assays that cover several key events of neural development in BrainSpheres we were able to detect alterations in neurite outgrowth, reduction of synaptic marker expression and a decrease in the number of oligodendrocytes after exposure to paroxetine at relevant therapeutic concentrations. These results identify paroxetine as a potential human developmental neurotoxicant, and suggest that the contraindication for its use should be evaluated and possibly extended far beyond the first trimester of pregnancy. In addition, we show that BrainSpheres allow to cover different aspects of brain development in one single system and constitute a novel tool to study and identify potential developmental neurotoxicants among chemicals and drugs, before their entry to the market.

## Data Availability Statement

The datasets generated for this study are available on request to the corresponding author.

## Author Contributions

XZ: western blots, stainings, and some cultures. GH: neurite outgrowth. LS: neurite outgrowth, immunohistochemistry, and supervision XZ, VZ, and MC: myelin and oligodendrocytes quantification. M-GZ: oligodendrocytes quantification, writing and revision of the manuscript. RS: BS cultures, viability assays, and mitochondrial function. FB and PB: synapsis quantification. PB: Synapsis quantification. HH: revision of the manuscript. TH: project idea, PI funding, head of laboratory, and revision of the manuscript. DP: cultures, immunohistochemistry, neurite outgrowth analysis, statistical analysis, coordinator of the experiments, and writer of the manuscript.

## Conflict of Interest

TH, HH and DP are named inventors on a patent by Johns Hopkins University on the production of mini-brains, which is licensed to AxoSim, New Orleans, LA, USA. They consult AxoSim and TH is shareholder. The remaining authors declare that the research was conducted in the absence of any commercial or financial relationships that could be construed as a potential conflict of interest.

## Footnotes

^1^https://imagej.nih.gov/ij/

^2^https://imagej.net/Sholl_Analysis

## Supplementary Material

FIGURE S1 BrainSpheres oligodendrocytes quantification panel. Confocal imagines of O4 positive marker for the four different experiments used for quantification.

FIGURE S2 Myelin quantification assay. BrainSphere myelination was quantified using a protocol adapted from Kerman et al. (2015) for computer-assisted evaluation of myelin formation on ImageJ. Myelination, is defined by the pixels overlapping between binary single-channel images of MBP staining and axons NF200 staining. **(A)** CEM quantification of one independent experiment (10 individual spheroids per condition) with the CLR–2097 line. The X-axis represents the concentration of paroxetine while the Y-axis represents the % of myelinated axons quantified by CEM plug-in. **(B)** Representative pictures of binary overlap between NF200 and MBP used for quantification. **(C)** Representative picture of BrainSpheres for neuronal marker (NF200) and myelin marker (MBP). Statistical analysis was performed by using ANOVA with Dunnett’s post-test comparing treated with control (untreated). **P* < 0.01.

FIGURE S3 Gene expression of SLC6A4. BrainSphere gene expression of SLC6A4 was quantified using qPCR. Housekeeping was Actin. No statistically significant changes were found.
