# Supplementary figures and images for: Antidepressant Paroxetine Exerts Developmental Neurotoxicity in an iPSC-Derived 3D Human Brain Model
Source: Front Cell Neurosci. 2020 Feb 21;14:25. doi: 10.3389/fncel.2020.00025 (PMC7047331; doi:10.3389/fncel.2020.00025)

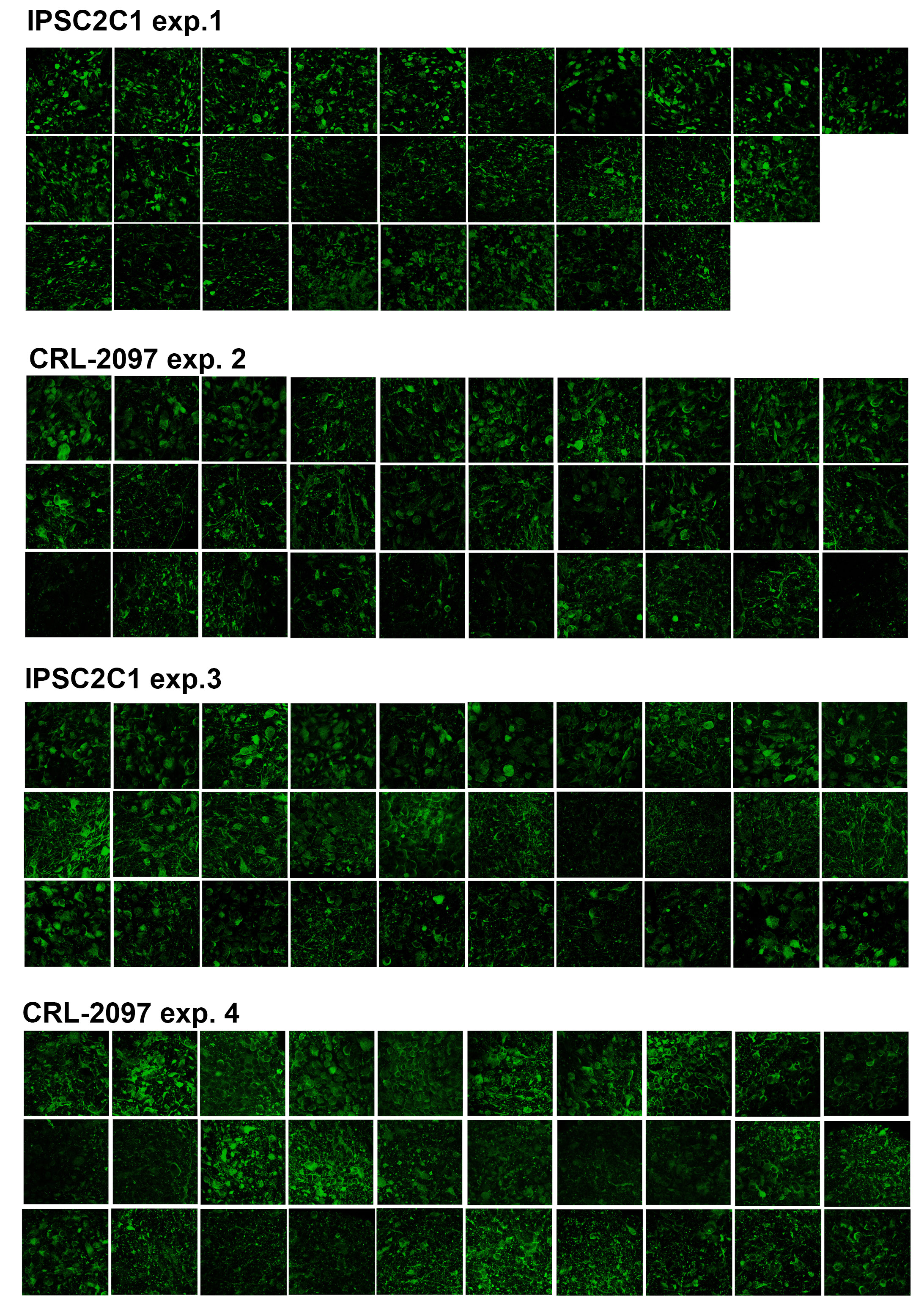

Supplement: FIGURE S1 — BrainSpheres oligodendrocytes quantification panel. Confocal imagines of O4 positive marker for the four different experiments used for quantification. [file Image_1.jpg]

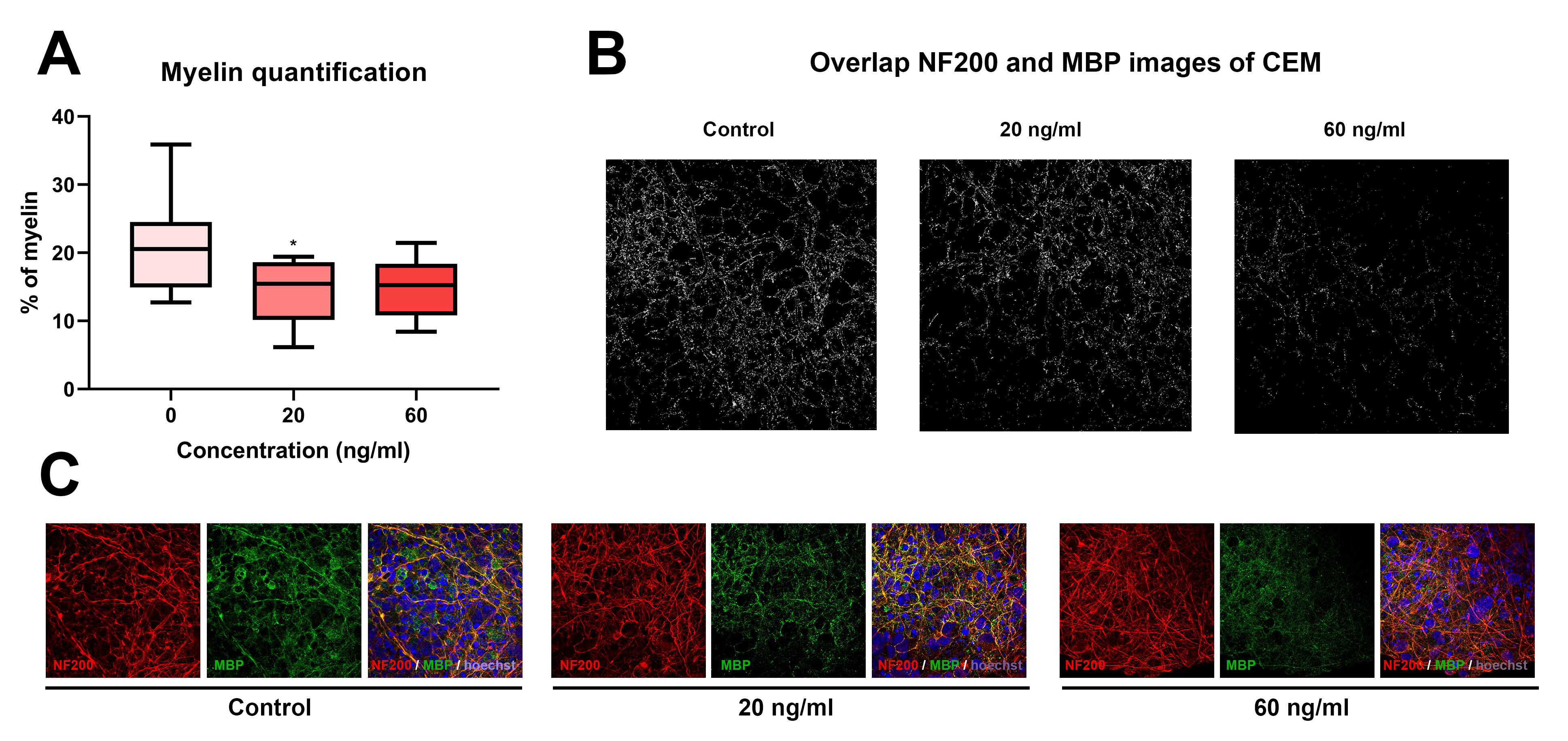

Supplement: FIGURE S2 — Myelin quantification assay. BrainSphere myelination was quantified using a protocol adapted from Kerman et al. (2015) for computer-assisted evaluation of myelin formation on ImageJ. Myelination, is defined by the pixels overlapping between binary single-channel images of MBP staining and axons NF200 staining. (A) CEM quantification of one independent experiment (10 individual spheroids per condition) with the CLR–2097 line. The X-axis represents the concentration of paroxetine while the Y-axis represents the % of myelinated axons quantified by CEM plug-in. (B) Representative pictures of binary overlap between NF200 and MBP used for quantification. (C) Representative picture of BrainSpheres for neuronal marker (NF200) and myelin marker (MBP). Statistical analysis was performed by using ANOVA with Dunnett’s post-test comparing treated with control (untreated). *P < 0.01. [file Image_2.JPEG]

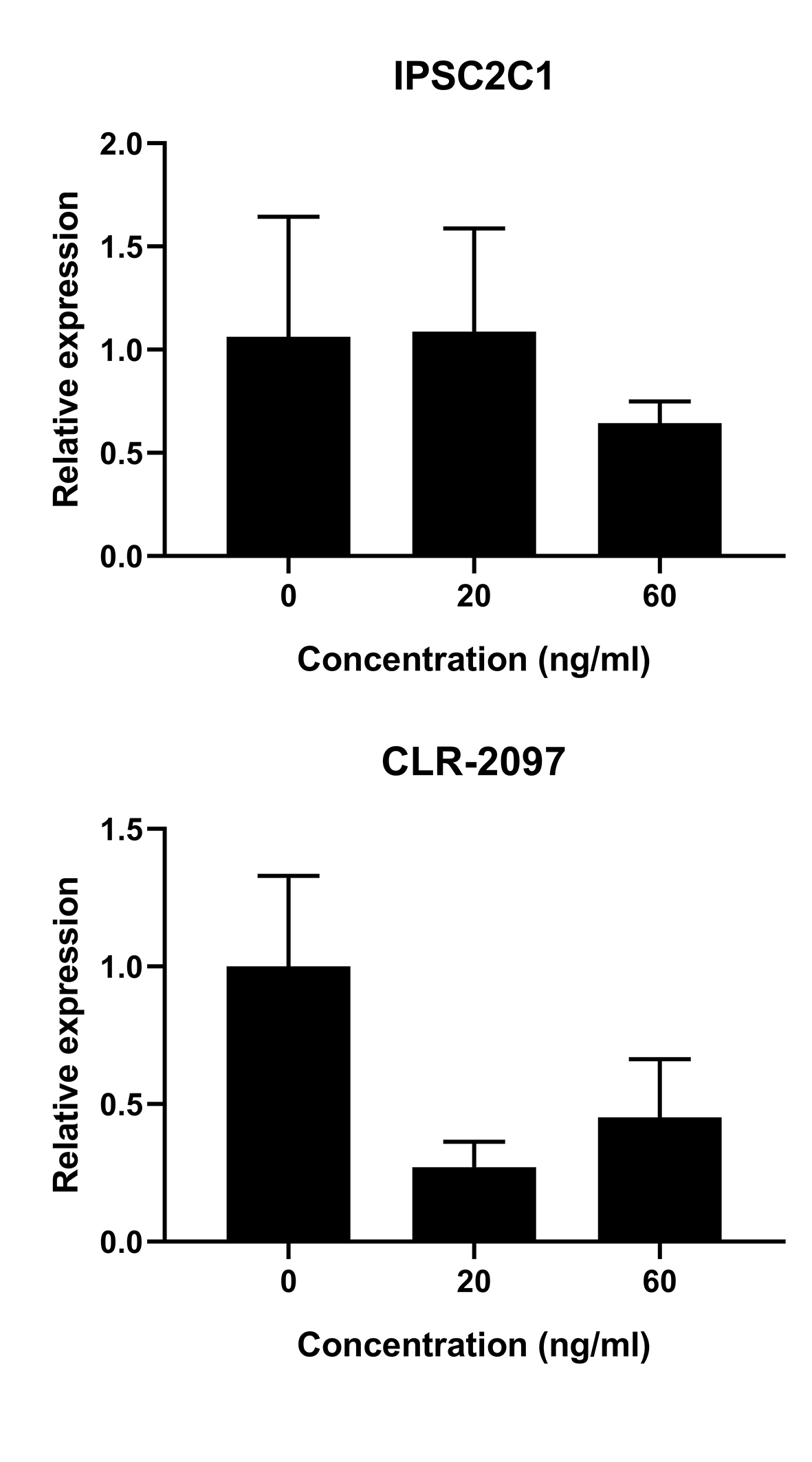

Supplement: FIGURE S3 — Gene expression of SLC6A4. BrainSphere gene expression of SLC6A4 was quantified using qPCR. Housekeeping was Actin. No statistically significant changes were found. [file Image_3.JPEG]
